# Dietary zinc intake is inversely associated with the risk of hypertension in the periodontitis population

**DOI:** 10.3389/fnut.2025.1616989

**Published:** 2025-07-15

**Authors:** Yvning Zhang, Yueyue Zhao, Yilu Zhong, Rui Zeng, Dongmei Ye, Dawei Guo, Wei Li

**Affiliations:** ^1^Affiliated Hospital of Jinggangshan University, School of Clinical Medicine, Jinggangshan University, Ji’an, Jiangxi, China; ^2^Renshou Center for Disease Control and Prevention, Renshou, Sichuan, China; ^3^Clinical Medical Research Center, Affiliated Hospital of Jinggangshan University, Ji’an, Jiangxi, China; ^4^Jiangxi Province Key Laboratory of Organ Development and Epigenetics, Ji’an, Jiangxi, China

**Keywords:** cardiovascular disease, dietary zinc, periodontitis, hypertension, oxidation, immune function

## Abstract

**Background:**

Periodontitis is a common chronic inflammatory disease, which is closely related to the development of several chronic diseases, including hypertension. The aim of this study was to investigate the association between dietary zinc intake and the risk of hypertension in a periodontitis population.

**Methods:**

We used a cross-sectional study design to select 10,061 participants from the National Health and Nutrition Examination Survey (NHANES) 2009–2014. The diagnosis of periodontitis was based on measurements of periodontal probing depth and clinical attachment loss. Dietary zinc intake was assessed using a 24-h dietary review survey. We used logistic regression analysis to assess the relationship between dietary zinc intake and hypertension, and stratified analysis and interaction tests to investigate the relationship between dietary zinc and hypertension in groups such as gender, ethnicity, and education.

**Results:**

Among United States adults with periodontitis, the risk of developing hypertension decreased by 1% for every 1 mg increase in daily dietary zinc intake (OR = 0.99, *p* = 0.011). Also, we found that high dietary zinc intake was associated with a lower risk of hypertension (OR = 0.84, *p* = 0.015).

**Conclusion:**

This study provides evidence that dietary zinc intake reduces the risk of hypertension in periodontitis patients. These findings suggest that monitoring and optimizing zinc nutritional status in periodontitis populations is important for hypertension prevention and treatment.

## 1 Introduction

Periodontitis is a common chronic inflammatory disease that is often caused by dysbacteriosis of the oral microbial flora. In severe cases, it leads to tooth loss in adults (1, 2). Many observational studies have shown that periodontitis is closely related to the progression of many chronic diseases in addition to its effects on periodontal tissues (3). Furthermore, there is increasing evidence that periodontitis may be associated with an increased risk of hypertension (4, 5). Consequently, it is crucial to focus on the correlation between periodontitis and hypertension and identify modifiable influencing factors in order to alleviate the disease burden of both periodontitis and hypertension.

Hypertension is a common chronic non-communicable disease that impacts people ’s health around the world and causes 10.4 million deaths each year (5). A 2021 American Heart Association report that hypertension was the second leading risk factor for loss of life in the United States in 1990 and 2019 (6). Hypertension not only has high morbidity and mortality, but also severely consumes medical and social resources and places a heavy financial burden on families and society (7). It is widely recognized that arterial hypertension constitutes a significant risk factor for cardiovascular disease. Moreover, the progression of periodontitis can result in elevated blood pressure levels and heighten the risk of developing arterial hypertension (8). Additionally, periodontitis may contribute to the ineffectiveness of antihypertensive treatments (9).

Periodontitis may exert effects on blood pressure through the systemic generalization of local oral inflammation, the role of host immune responses, direct microbial effects on the vascular system, and alterations in endothelial function (10). This was primarily linked to low-grade systemic inflammation, which is associated with poor periodontal health. This connection was further evidenced by the reduction in systemic pro-inflammatory factors linked to hypertension after non-surgical periodontal therapy in patients with periodontitis (11). As a versatile trace element, zinc can bind more than 300 enzymes and more than 2,000 transcription factors and is one of the most important micronutrients involved in many key biological functions (12). Zinc can modulate immune responses and possess antioxidant/anti-inflammatory activities, which exert antioxidant effects by inducing expression of metallothionein and anti-inflammatory effects by increasing expression of anti-inflammatory genes (13). Increased inflammation caused by zinc deficiency releases more inflammatory mediators and can adversely affect periodontitis (14). Therefore, adequate zinc intake may help to reduce the risk of periodontitis while reducing the risk of hypertension. Previous studies have shown that imbalances in serum levels of zinc, copper, and iron predispose individuals to the risk of periodontitis, with diabetic patients with periodontitis having decreased serum zinc levels and increased serum iron and copper levels compared to healthy individuals with and without periodontitis (15–17). However, no study has pointed out the relationship between serum zinc and hypertension in periodontitis patients. Therefore, based on the mechanism of the immune inflammatory response in humans, we hypothesize that people with periodontitis have an increased risk of hypertension when zinc is deficient, and conversely a decreased risk of hypertension. The main objective of our study was to investigate the relationship between dietary zinc and hypertension in a periodontitis-affected population based on National Health and Nutritional Examination Survey (NHANES) from 2009 to 2014.

## 2 Materials and methods

### 2.1 Study design and data source

Data for this cross-sectional study came from NHANES 2009–2014. NHANES is a nationally representative publicly available database designed to assess the health and nutritional status of adults and children in the United States. The survey uses a stratified multistage sampling design and weighting scheme to select representative samples from the United States population, combined with interviews and field visits, and surveys a nationally representative sample of about 5,000 people each year. More detailed information about the NHANES database can be found here^1^. A total of 30,648 people participated in NHANES from 2009 to 2014, however, after applying the exclusion criteria, 10,061 periodontitis patients aged ≥ 30 years were included in this study. Exclusion criteria included: (1) absence of periodontal data (*n* = 19,069); (2) absence of hypertension data (1); (3) absence of dietary zinc data (*n* = 690); (4) removal of dietary zinc extremes (*n* = 1); and (5) exclusion of people without periodontitis (*n* = 646).

### 2.2 Periodontitis

Periodontal probing depth (PD) and clinical attachment loss (AL) were measured by trained examiners performing a comprehensive and standardized periodontal examination at six locations per tooth in the full mouth (excluding third molars). Periodontal examiners received high intensity training and were then monitored regularly and recalibrated according to the reference examiner. The reference examiner makes three visits to each dental examination team each year to observe field operations and perform replicate 20–25 oral health examinations. Periodontitis was classified using the 2018 World Symposium classification system and was diagnosed when interdental AL was ≥ 1 mm in ≥ 2 non-adjacent teeth or AL was ≥ 3 mm in buccal/lingual sites and PD was ≥ 3 mm in ≥ 2 teeth (18).

### 2.3 Dietary zinc intake

National Health and Nutrition Examination Survey’ dietary zinc intake and its supplement intake were based on two 24 h dietary recall surveys. All participants underwent two 24 h dietary recall interviews, the first conducted in person by trained interviewers at a mobile screening center (MEC), and the second dietary recall interview collected by phone or mail 3–10 days later. In our study, we used transcripts of the first 24 h diet recall interview to minimize recall bias (19).

### 2.4 High blood pressure

In NHANES, blood pressure measurements were obtained by trained clinicians using a mercury sphygmomanometer with appropriately sized cuffs. Participants rested in a seated position for 5 min before three consecutive readings were taken at 30 s intervals, with the average of these measurements used for analysis. Hypertension was defined as meeting any one of the following criteria: (1) Systolic blood pressure (SBP) ≥ 130 mmHg or diastolic blood pressure (DBP) ≥ 80 mmHg, based on examination data from NHANES (20). (2) The presence of self-reported hypertension was determined based on the questionnaire item: “Have you/ever been told by a doctor or other health professional that you have high blood pressure, also called hypertension?” (3) The use of antihypertensive medications was assessed through the question: “Prescription drug now being taken?” (21).

### 2.5 Assessment of covariates

We collected relevant covariates, including: sex, age, race, education, body mass index (BMI), serum iron, total cholesterol, high-density lipoprotein cholesterol (HDL), asthma, heart attack, angina pectoris, emphysema, coronary heart disease, gout, anemia, congestive heart failure, and salt intake. Gender was divided into males and females. Ethnicity was divided into Mexican Americans, other Hispanics, non-Hispanic whites, non-Hispanic blacks, and other races. Education was categorized as less than grade 9, grade 9–11 (including grade 12, no diploma), high school graduation/GED or equivalent, Some College or AA degree, and College Graduate or above. BMI was derived from the examination data in kg/m^2^ and was calculated by dividing weight by the square of height. Serum iron, total cholesterol, and high-density lipoprotein cholesterol were obtained from laboratory data. Diabetes was diagnosed as having one of the following criteria: (1) participants’ self-reported diagnosis of diabetes; (2) glycosylated hemoglobin (HbA1c) ≥ 6.5%; (3) fasting blood glucose (FBG) ≥ 7.0 mmol/L; and (4) a 2 h oral glucose tolerance test (OGTT) result ≥ 11.1 mmol/L (22). Smoking was divided into three categories: never smokers (less than 100 cigarettes smoked in a lifetime), past smokers (more than 100 cigarettes smoked in a lifetime and not smoked at all now), and current smokers (more than 100 months smoked in a lifetime and sometimes smoked every day) (23). Salt intake was defined by the question “How often do you use regular or seasoned salt when cooking or preparing your food” in the dietary data. Asthma, heart attack, angina pectoris, emphysema, coronary heart disease, gout, anemia, congestive heart failure, can be found in questionnaire data.

### 2.6 Statistical analyses

This study used a cross-sectional study design. Normality tests were performed to assess the distribution of continuous variables. All continuous variables are presented as mean (standard deviation) (SE), and all categorical variables are presented as number (percentage). The characteristics of the groups of hypertensive subjects and non-hypertensive subjects were compared using *t*-test and Chi-square test (Table 1). We handled missing covariates as follows: NHANES data for years 09–14 were multiply imputed with Rstudio (version 4.4.1) to improve completeness of data and reduce estimation bias. Stratified analysis and interaction tests were used to investigate the relationship between dietary zinc and hypertension in groups such as gender, ethnicity, and education, and confounding factors such as: age, gender, ethnicity, education, household income poverty ratio, serum iron, total cholesterol, high-density lipoprotein cholesterol, alcohol consumption, heart attack, angina pectoris, emphysema, coronary heart disease, gout, arthritis, anemia, congestive heart failure, salt intake, and smoking were adjusted (Table 2). Logistic regression was used to assess the relationship between dietary zinc and hypertension, and three models were constructed, model one was unadjusted, model two was adjusted according to gender, age, race, education, and family income to poverty income ratio, and model three adjusted the components of model two plus serum iron, alcohol consumption, total cholesterol, high-density lipoprotein cholesterol, congestive heart failure, heart attack, angina pectoris, emphysema, coronary heart disease, gout, arthritis, anemia, smoking, and salt intake. In addition, subgroup analysis was performed to further analyze the relationship between asthma, dietary zinc in body mass index and hypertension to test the robustness of the model (Table 3). *P*-values for trend were calculated using the median of quartiles as a quasi-continuous variable in the model (Table 3). Restrictive cubic spline (RCS) analysis was used to test the non-linear relationship between dietary zinc and hypertension. All data were analyzed using Rstudio (version 4.4.1) and Empowerstats. Data were considered statistically significant when *P* < 0.05.

**TABLE 1:** Characteristics of the participants (*N* = 10,061).

Hypertension recoded	Overall^1^	No hypertension	Hypertension	*P* ^2^
*N*	10,061	4,583	5,478	–
Age	–	–	–	<0.001
<65	7,930 (78.82%)	4,149 (90.53%)	3,781 (69.02%)	–
≥65	2,131 (21.18%)	434 (9.47%)	1,697 (30.98%)	–
Gender	–	–	–	<0.001
Male	4,959 (49.29%)	2,094 (45.69%)	2,865 (52.30%)	–
Female	5,102 (50.71%)	2,489 (54.31%)	2,613 (47.70%)	–
The ratio of household income to poverty	2.65 ± 1.62	2.72 ± 1.65	2.59 ± 1.59	<0.001
Serum iron	84.31 ± 33.77	85.73 ± 34.23	83.12 ± 33.34	<0.001
Dietary magnesium	305.76 ± 151.53	316.91 ± 158.20	296.43 ± 145.06	<0.001
Dietary copper	1.30 ± 1.07	1.34 ± 1.12	1.26 ± 1.02	<0.001
Dietary iron	14.83 ± 8.51	15.11 ± 8.57	14.60 ± 8.46	<0.001
Dietary zinc	11.21 ± 6.69	11.50 ± 6.66	10.96 ± 6.71	<0.001
Total cholesterol	198.02 ± 41.13	197.96 ± 39.17	198.07 ± 42.71	0.987
High density lipoprotein cholesterol	52.89 ± 15.90	53.83 ± 15.70	52.11 ± 16.02	<0.001
Body mass index	–	–	–	<0.001
<30	6,087 (60.50%)	3,203 (69.89%)	2,884 (52.65%)	–
≥30	3,974 (39.50%)	1,380 (30.11%)	2,594 (47.35%)	–

^1^Continuous variables are the mean (standard deviation), and other categorical variables are the number of people (percentage).

^2^*t*-test for continuous variables and chi-square test for classified variables.

**TABLE 2:** Relationship between hypertension and serum zinc in categorical variables.

Participants	*N*	OR (95% CI)	*P*-value	*P* for interaction
Dietary zinc	–	–	–	–
Gender	–	–	–	0.846
Male	4,959 (49.29%)	0.99 (0.98, 1.00)	0.028	–
Female	5,102 (50.71%)	0.99 (0.98, 1.00)	0.199	–
Drink	–	–	–	0.478
Yes	7,434 (73.89%)	0.99 (0.99, 1.00)	0.048	–
No	2,627 (26.11%)	0.99 (0.97, 1.00)	0.072	–
Asthma	–	–	–	0.042
Yes	1,372 (13.64%)	0.97 (0.96, 0.99)	0.005	–
No	8,689 (86.36%)	0.99 (0.99, 1.00)	0.089	–
Congestive heart-failure	–	–	–	0.777
Yes	217 (2.16%)	0.98 (0.92, 1.05)	0.591	–
No	9,844 (97.84%)	0.99 (0.99, 1.00)	0.013	–
Heart attack	–	–	–	0.683
Yes	311 (3.09%)	1.00 (0.96, 1.04)	0.989	–
No	9,750 (96.91%)	0.99 (0.98, 1.00)	0.010	–
Angina	–	–	–	0.250
Yes	190 (1.89%)	1.03 (0.96, 1.10)	0.392	–
No	9,871 (98.11%)	0.99 (0.98, 1.00)	0.009	–
Stroke	–	–	–	0.229
Yes	278 (2.76%)	1.03 (0.97, 1.10)	0.379	–
No	9,783 (97.24%)	0.99 (0.98, 1.00)	0.008	–
Emphysema	–	–	–	0.649
Yes	146 (1.45%)	0.98 (0.94, 1.02)	0.400	–
No	9,915 (98.55%)	0.99 (0.99, 1.00)	0.015	–
Coronary heart disease	–	–	–	0.887
Yes	304 (3.02%)	0.99 (0.96, 1.03)	0.770	–
No	9,757 (96.98%)	0.99 (0.98, 1.00)	0.012	–
Gout	–	–	–	0.225
Yes	418 (4.15%)	1.02 (0.98, 1.06)	0.452	–
No	9,643 (95.85%)	0.99 (0.98, 1.00)	0.007	–
Arthritis	–	–	–	0.910
Yes	2,720 (27.04%)	0.99 (0.98, 1.01)	0.287	–
No	7,341 (72.96%)	0.99 (0.98, 1.00)	0.021	–
Anemia	–	–	–	0.113
Yes	386 (3.84%)	1.02 (0.98, 1.06)	0.272	–
No	9,675 (96.16%)	0.99 (0.98, 1.00)	0.006	–
Salt intake	–	–	–	0.837
Never	786 (7.81%)	1.00 (0.98, 1.02)	0.942	–
Rarely	1,820 (18.09%)	0.99 (0.98, 1.01)	0.229	–
Occasionally	3,223 (32.03%)	0.99 (0.98, 1.00)	0.151	–
Very often	4,232 (42.06%)	0.99 (0.98, 1.00)	0.041	–
Smoke	–	–	–	0.433
Never	5,643 (56.09%)	0.99 (0.98, 1.00)	0.047	–
Formerly	2,534 (25.19%)	1.00 (0.99, 1.01)	0.750	–
Now	1,884 (18.73%)	0.99 (0.97, 1.00)	0.038	–
Body mass index	–	–	–	0.997
<30	6,087 (60.50%)	0.99 (0.98, 1.00)	0.032	–
≥30	3,974 (39.50%)	0.99 (0.98, 1.00)	0.088	–
Age	–	–	–	0.841
<65	7,930 (78.82%)	0.99 (0.98, 1.00)	0.022	–
≥65	2,131 (21.18%)	0.99 (0.97, 1.01)	0.232	–
Diabetes	–	–	–	0.626
Yes	8,227 (81.77%)	0.99 (0.98, 1.00)	0.013	–
No	1,834 (18.23%)	1.00 (0.98, 1.01)	0.583	–

The calculation of *P* for Interaction adjusts: gender, age, racial, education, the ratio of household income to poverty, serum iron, drink, total cholesterol, high density lipoprotein cholesterol, congestive heart failure, heart attack, angina pectoris, emphysema, coronary heart disease, gout, arthritis, anemia, smoke, salt intake.

**TABLE 3:** Results of logistic regression and trend test.

Exposure	Model I*		Model II*		Model III*	
	OR (95% CI)	*P*-value	OR (95% CI)	*P*-value	OR (95% CI)	*P*-value
Dietary zinc	0.99 (0.98, 0.99)	<0.001	0.99 (0.98, 1.00)	0.002	0.99 (0.99, 1.00)	0.011
Dietary zinc quartile	–	–	–	–	–	–
Q1	1.0	–	1.0	–	1.0	–
Q2	0.91 (0.80, 1.03)	0.119	0.92 (0.80, 1.05)	0.216	0.93 (0.81, 1.07)	0.302
Q3	0.83 (0.73, 0.94)	0.004	0.91 (0.79, 1.04)	0.157	0.92 (0.80, 1.06)	0.238
Q4	0.78 (0.68, 0.88)	<0.001	0.85 (0.74, 0.97)	0.018	0.86 (0.75, 0.99)	0.035
Q5	0.76 (0.67, 0.86)	<0.001	0.81 (0.71, 0.93)	0.003	0.84 (0.73, 0.97)	0.015
Trend test of five groups of dietary zinc	0.98 (0.97, 0.99)	<0.001	0.99 (0.98, 1.00)	0.003	0.99 (0.98, 1.00)	0.012
Asthma	–	–	–	–	–	–
Yes	0.97 (0.96, 0.99)	<0.001	0.98 (0.96, 0.99)	0.011	0.98 (0.96, 1.00)	0.028
No	0.99 (0.98, 1.00)	0.002	0.99 (0.98, 1.00)	0.019	0.99 (0.99, 1.00)	0.053
Body mass index	–	–	–	–	–	–
<30	0.99 (0.98, 1.00)	0.006	0.99 (0.98, 1.00)	0.008	0.99 (0.98, 1.00)	0.038
≥30	0.99 (0.98, 1.00)	0.008	0.99 (0.98, 1.00)	0.040	0.99 (0.98, 1.00)	0.053

Non-adjusted model adjust for: none. Model I*: non-adjusted. Model II*: adjust for, gender, age, racial, education, the ratio of household income to poverty. Model III*: adjust for, gender, age, racial, education, the ratio of household income to poverty, serum iron, drink, total cholesterol, high density lipoprotein cholesterol, congestive heart failure, heart attack, angina pectoris, emphysema, coronary heart disease, gout, arthritis, anemia, smoke, salt intake.

## 3 Results

### 3.1 Selection and characteristics of patients

**FIGURE 1:**
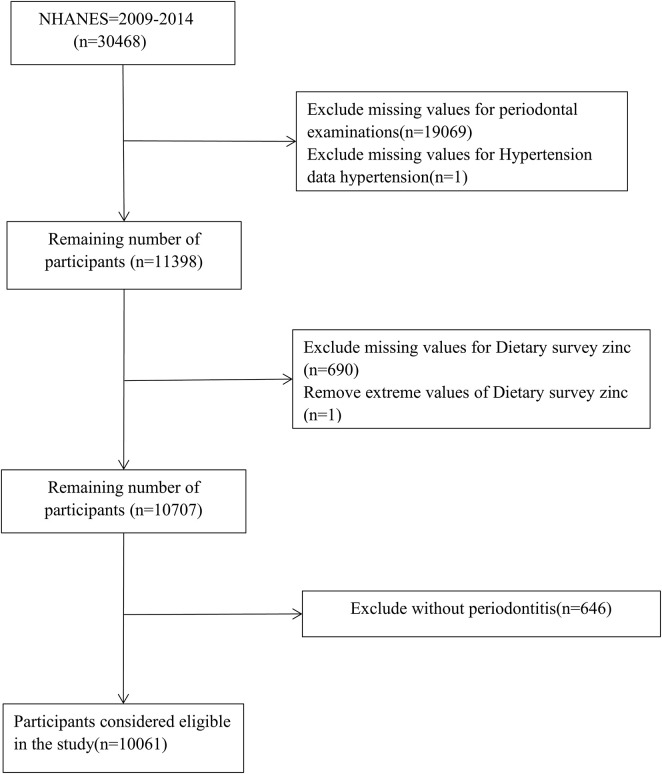
Participants flowchart.

Figure 1 showed the study recruitment and inclusion/exclusion criteria for this study and ultimately 10,061 participants from the NHANES database were included in the analysis. Table 1 displays the subjects’ baseline characteristics, stratified by hypertension status. In our study, 5,478 participants had both periodontitis and hypertension, and 4,583 participants had only periodontitis. Those with hypertension had lower dietary zinc intake and HDL levels than those without hypertension, and they were more likely to had higher body mass index and total cholesterol (Table 1).

### 3.2 Stratified assessment and interaction of categorical variables

The stratified analyses of the associations between dietary zinc intake and the risk of hypertension were presented in Table 2. Asthma patients exhibited a strong association between zinc intake and hypertension (OR= ~ 0.97, *P*= ~ 0.005). Interaction analysis showed that the ORs between dietary zinc intake and the risk of hypertension was lower in the asthma group than in the non-asthma group (*P* for interaction = 0.042).

### 3.3 Association between dietary zinc intake and risk of hypertension

The relationship between dietary zinc and hypertension was shown in Table 3. Logistic regression analysis showed that dietary zinc intake was significantly negatively associated with hypertension in periodontitis patients regardless of confounding factors adjustment. Adults with periodontitis in the United States have a 1% lower risk of hypertension for every milligram increase in daily dietary zinc intake (OR = 0.99, *P* = 0.011). Dietary zinc intake was further divided into quintiles, and Q1 group was used as the reference group to evaluate the relationship between dietary zinc intake and hypertension. After adjusting for gender, age, racial, education, the ratio of household income to poverty, serum iron, drink, total cholesterol, high density lipoprotein cholesterol, congestive heart failure, heart attack, angina pectoris, emphysema, coronary heart disease, gout, arthritis, anemia, smoke, and salt intake, we found that people with daily zinc intake greater than 15 mg had a 16% lower risk of hypertension compared with those with daily zinc intake less than 6 mg (OR = 0.84, *P* = 0.015, *P* = 0.012 for trend). We observed similar results in the subgroup of asthmatic patients and in the subgroup with a body mass index less than 30 (OR = 0.98, *P* = 0.028, *P* for interaction = 0.042; OR = 0.99, *P* = 0.038, *P* for interaction = 0.997).

### 3.4 Dose–response effect examination

**FIGURE 2:**
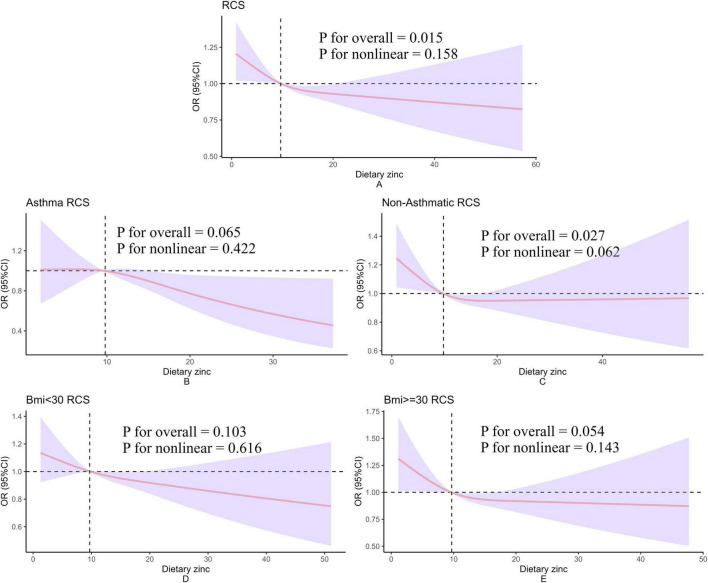
Restricted cubic spline model of hypertension and dietary zinc intake. All of these were adjusted for gender, age, racial, education, the ratio of household income to poverty, serum iron, drink, total cholesterol, high density lipoprotein cholesterol, asthma, congestive heart failure, heart attack, angina pectoris, emphysema, coronary heart disease, gout, arthritis, anemia, smoke, salt intake. **(A–E)** Present the dose-response relationships between dietary zinc intake and hypertension analyzed using restricted cubic splines (RCS) in the overall population, asthma population, non-asthma population, BMI < 30 group, and BMI ≤ 30 group, respectively. The corresponding subgroup labels are indicated in the top-left corner of each panel. The y-axis label “OR (95% CI)” denotes the odds ratio and its 95% confidence interval. The x-axis label “Dietary zinc” represents daily dietary zinc intake. “P for nonlinear” refers to the *p*-value for testing nonlinear association, while “P for overall” indicates the *p*-value for testing the overall association.

The dose-response relationship between dietary zinc intake regressed by RCS and hypertension is shown in Figure 2. The overall population dose-response test indicated that dietary zinc intake was linearly and inversely associated with the risk of hypertension (Figure 2A). At the same time, we did asthma and body mass index subgroups, but no significant association was found (Figures 2B–E).

## 4 Discussion

The current study explored the dose–response relationship between dietary zinc intake and the risk of hypertension. A linear inverse correlation was identified, indicating that for every 1 mg increase in daily dietary zinc consumption, there was a corresponding 1% decrease in the risk of hypertension among United States adults suffering from periodontitis.

To verify the robustness of the results, we performed a trend test. Using the lowest quintile of dietary zinc intake as a reference, the results showed that the risk of hypertension significantly decreased with increasing dietary zinc intake. A 16% reduction in hypertension risk was observed among participants with daily zinc intake exceeding 15 mg relative to those with intake under 6 mg (OR = 0.84, *P* = 0.015, *P* = 0.012 for trend). People with higher zinc intake was significantly linked to a lower risk of hypertension.

We observed an inverse association between dietary zinc intake and risk of hypertension in stratified analyses with body mass index less than 30 kg/m^2^. We explored body mass index more deeply. The results showed that zinc intake in asthmatics reduces the risk of hypertension. We also found that dietary zinc was inversely associated with the risk of hypertension in people with a body mass index below 30 kg/m^2^. However, a previous study showed that serum zinc concentration in normal weight was positively associated with the risk of hypertension (24). This contradicts our results and may be due to factors such as different biological markers, population differences, methodological heterogeneity, etc., Further studies are needed to investigate the potential impact of body mass index and asthma.

In this study, we investigated the complex relationship between zinc deficiency and hypertension, periodontitis and immune function, revealing the critical role of inflammation as a common pathway. Zinc ranks as the second most prevalent transition metal within the human body, following iron, and a deficiency in zinc can impact the development of numerous organs, such as the heart, brain, lungs, kidneys, and bones (25). Although the body possesses the ability to maintain zinc levels within a normal range, factors such as low intake, malabsorption, and heightened losses within the gastrointestinal system collectively contribute to zinc deficiency (26). The effect of zinc on immune function has been well documented, and zinc deficiency affects a variety of immune cell types, especially the thymus, making the body more susceptible to microbial interference and increasing susceptibility to disease, leading to the progression of chronic and degenerative diseases, namely type 2 diabetes mellitus (T2DM), cardiovascular disease (CVD), and cancer (27). Immune dysfunction due to zinc deficiency is significantly associated with increased levels of pro-inflammatory cytokines, which may be an important mechanism linking periodontitis and hypertension (28). Available evidence suggests multiple roles for zinc in maintaining vascular homeostasis. For example, zinc affects blood pressure regulation by regulating vascular smooth muscle tension, and its antioxidant properties protect vascular endothelium from oxidative stress injury, and endothelial dysfunction is an important mechanism of the development of hypertension (12). Insufficient dietary zinc intake has been shown to alter individual taste sensitivity to salt, resulting in increased salt intake, a well-known risk factor for hypertension (29), as demonstrated in an animal study (30). Therefore, low zinc or zinc deficiency is likely to be the cause of hypertension, and our results do suggest that low zinc is associated with a high risk of hypertension. Meanwhile, in our study, the study subjects had a distinct feature: periodontitis. The results of a systematic evaluation indicated that patients with moderate to severe periodontitis had a higher likelihood (20%) of also having hypertension compared to those without periodontitis (31). Periodontitis affects endothelial function through systemic inflammation involving mediators such as CRP, IL-6, and TNF-α that can affect endothelial function (31), which may contribute to hypertension development. This is consistent with Tonetti et al. ‘s findings that treating severe periodontitis improves endothelial function by reducing systemic inflammation in patients regardless of whether they have other comorbidities (32). Therefore, it is important to treat periodontitis, a common disease, for protecting cardiovascular structure. Previous findings support a bidirectional relationship between periodontitis and zinc deficiency. On the one hand, zinc deficiency promotes periodontitis progression by compromising oral mucosal integrity, weakening local immune defenses, and promoting the secretion of pro-inflammatory cytokines (33); on the other hand, chronic inflammatory states triggered by periodontitis may further exacerbate zinc metabolism disorders (34). Thus, inflammation plays a central role in diseases associated with zinc deficiency–zinc deficiency promotes the release of pro-inflammatory factors that impair both vascular function and periodontal tissues (14, 35). A cross-sectional study has shown the moderating effect of sufficient zinc intake on the association between periodontitis and ASCVD, providing guidance for reducing ASCVD risk in periodontitis patients (36). In summary, zinc deficiency in periodontitis populations impacts an individual’s risk of hypertension by affecting systemic inflammation, and adequate zinc intake may assist in lowering the risk of both periodontitis and hypertension, and this population should receive professional oral care and dietary advice to reduce or prevent the risk of hypertension. This therefore this suggests that clinicians need to pay more attention to zinc intake in periodontitis people to avoid possible hypertension outcomes. At the same time, our study provides ideas for subsequent experimental studies.

This study showed several notable advantages. First, our study is the first to investigate the effect of dietary zinc on hypertension based on a periodontitis population. Second, this study is based on data from NHANES, which uses a stratified multistage sampling design and weighting scheme to select samples that are well representative of the United States non-institutional population. And the analysis was performed on a larger sample size, therefore, the conclusions of this study should be applicable to the general population in the United States. In addition, we performed multiple imputation of the data to improve completeness of the data. Nevertheless, our article does have its limitations. Although the study’s findings indicated a noteworthy correlation between dietary zinc intake and hypertension, it is important to note that this research was of a cross-sectional nature. Consequently, we cannot ascertain a causal link between dietary zinc and hypertension. Second, even though we adjusted for confounding factors, at the same time, we also discuss the possible curve relationships and do subgroup analysis. In addition, we have carried out several sensitivity analyses, and the results have good robustness. We still could not completely exclude the potential influence of confounding factors. Third, this study covers individuals over 30 years of age and further research is needed to determine whether the findings apply to younger populations. Ultimately, the sample population for this study was drawn from the United States, which could restrict the broader relevance of the results to the global population. Future research should take into account larger and more diverse groups to establish effective dietary zinc thresholds for managing hypertension.

## 5 Conclusion

This research indicates that higher dietary zinc consumption is inversely correlated with the incidence of hypertension among United States adults suffering from periodontitis. Augmenting zinc intake could serve as a potential strategy to prevent hypertension in adult patients with periodontitis in the United States.

## Data Availability

The original contributions presented in this study are included in this article/supplementary material, further inquiries can be directed to the corresponding authors.

## Footnotes

1
https://wwwn.cdc.gov/nchs/nhanes/Default.aspx
